# Computed modeling of alternating electric fields therapy for recurrent glioblastoma

**DOI:** 10.1002/cam4.519

**Published:** 2015-08-26

**Authors:** Edwin Lok, Van Hua, Eric T Wong

**Affiliations:** 1 Brain Tumor Center and Neuro-Oncology Unit, Department of Neurology, Harvard Medical School, Beth Israel Deaconess Medical CenterBoston, Massachusetts; 2 Massachusetts College of Pharmacy and Health Sciences UniversityBoston, Massachusetts; 3 Department of Physics, University of Massachusetts at LowellLowell, Massachusetts

**Keywords:** Electric fields, glioblastoma

## Abstract

Tumor treating fields (TTFields) are alternating electric fields frequency tuned to 200 kHz for the treatment of recurrent glioblastoma. We report a patient treated with TTFields and determined the distribution of TTFields intracranially by computerized simulation using co-registered postgadolinium T1-weighted, T2, and MP RAGE images together with pre-specified conductivity and relative permittivity values for various cerebral structures. The distribution of the electric fields within the brain is inhomogeneous. Higher field intensities were aggregated near the ventricles, particularly at the frontal and occipital horns. The recurred tumor was found distant from the primary glioblastoma and it was located at a site of relatively lower electric field intensity. Future improvement in TTFields treatment may need to take into account the inhomogeneity of the electric field distribution within the brain.

## Introduction

Alternating electric fields or tumor treating fields (TTFields) therapy is a novel treatment modality for recurrent glioblastoma 1. The TTFields are frequency tuned to 200 kHz and are delivered by the FDA-approved NovoTTF-100A System (Novocure, Inc., Haifa, Israel) via two pairs of orthogonally positioned arrays placed on the patient’s shaved scalp 1. In past clinical trial for recurrent glioblastomas, TTFields have been shown to have equivalent efficacy when compared to conventional chemotherapies, whereas for newly diagnosed glioblastomas TTFields plus temozolomide appear to have superior efficacy when compared to temozolomide alone in interim analysis 2,3. However, the precise distribution of TTFields within the brain, and the extent to which they cover the recurrent glioblastoma, remains poorly understood. Here, we report our analysis using computerized simulation to determine the frequency-dependent electric field distribution inside a patient’s brain, using co-registered postgadolinium T1-weighted, T2, and MP RAGE images together with prespecified conductivity and relative permittivity values for the cerebral structures (http://www.itis.ethz.ch/itis-for-health/tissue-properties/database/dielectric-properties/). We found that the electric fields on the scalp mirror that of the observed skin irritation. The strength of TTFields is highest at the ventricular horns and the medial surface of the tumor.

## Results and Discussion

**Figure 1:**
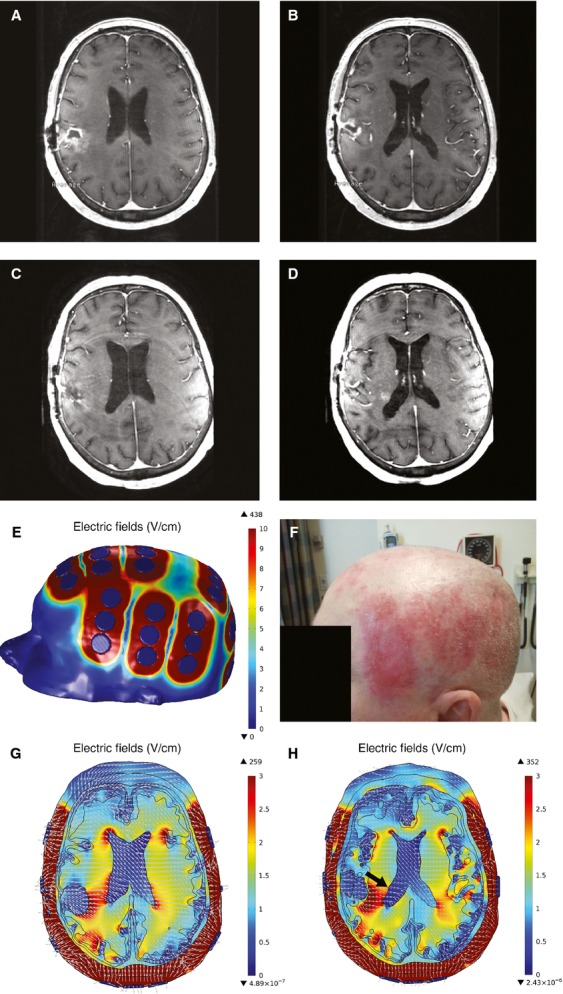
Tumor treating fields (TTFields) therapy for recurrent glioblastoma. Recurrent glioblastoma in the posterior right frontal brain was detected in a 67-year-old woman 6 months after initial treatment. The tumor can be seen in two successive slices, upper slice (A) and lower slice (B) on gadolinium-enhanced T1-weighted MP RAGE sequence. Her recurrent disease was then treated with bevacizumab and TTFields using the NovoTTF-100A device and after 24 months a new site of disease was detected at the lateral border of the right lateral ventricle (D), while the primary tumor was stable (C). Computed modeling revealed that the electric field strength was highest around the ceramic disks forming each array (E), which is also located at the same site of scalp irritation experienced by the patient (F). High field strength is also seen at the ventricular horns and there is inhomogeneous electric field distribution on the tumor (G). The new enhancing tumor is situated at the lateral border of the right lateral ventricle in an area with relatively lower electric field strength (H, arrow).

We performed an IRB-approved retrospective neuroimaging analysis of TTFields therapy in a 67-year-old woman with recurrent glioblastoma in the posterior right frontal brain (Fig.1A and B) 6 months after the initial treatment with neurosurgical resection, cranial irradiation with concomitant daily temozolomide, and followed by adjuvant temozolomide. She then received both bevacizumab, infused at a dose of 10 mg/kg every 2 weeks, and TTFields therapy, with which she applied onto her shaved head continuously when possible. The placement of the transducer arrays was based on a personalized computer-generated layout, which is derived from MRI-based morphometric measurements of her head, tumor size, and tumor location. She continued both treatments for 24 months until another site of disease was detected in the lateral border of the right lateral ventricle (Fig.1C and D).

From the same baseline MRI used for array layout, a three-dimensional rendition of the head was generated based on the co-registered MR image datasets using ScanIP software (Simpleware Ltd., Exeter, U.K.). A finite element mesh was generated for each of the segmented head structures including the scalp, skull, dura, cerebrospinal fluid (CSF), supratentorial gray/white matter, ventricles, brainstem, cerebellum, and the recurrent glioblastoma. The composite finite element mesh model was imported into COMSOL Multiphysics 5.0 (Burlington, MA) to solve for the electric field distribution. The respective conductivity and relative permittivity values used were 0.001 S/m and 1110 for scalp, 0.021 S/m and 204 for skull, 0.502 S/m and 290 for dura, 2.000 S/m and 109 for CSF, 0.141 S/m and 2010 for gray matter, 0.087 S/m and 1290 for white matter, and 1.000 S/m and 10,000 for recurrent glioblastoma 4.

This is the first electric field simulation of a patient with recurrent glioblastoma treated with TTFields therapy and our analysis revealed important information relevant to the clinical care of patients. First, the highest electric field strength was found around the ceramic disks forming each array (Fig.1E), which is also located at the same site of scalp irritation experienced by the patient (Fig.1F). Although the hydrogel situated between the ceramic disk and the scalp may cause local dermatologic irritation, the high intensity of the electric fields beneath the disks may be another contributor. This irritation can be reduced by shifting the arrays by 10–20 mm clockwise or counterclockwise when exchanged at 3–4 day intervals 5. Second, because water has high conductivity and low relative permittivity, the electric fields are expected to converge toward the ventricles. Miranda et al. 6 have shown that the highest field intensity aggregated at the periventricular region. Likewise, we observed the same phenomenon but the highest field strength is localized at the ventricular horns, most likely due to the large relative difference in dielectric properties and the inhomogeneity of the different tissues. Finally, we detected inhomogeneous electric field distribution on the tumor, with the medial border facing the right lateral ventricle having higher field strength than the lateral border (Fig.1G). Furthermore, this patient developed a new site of disease during TTFields therapy and her head MRI revealed a new enhancing tumor situated at the lateral border of the right lateral ventricle (Fig.1H, arrow), a location with relatively lower electric field strength. Tumor cell invasion into the surrounding brain may explain this phenomenon. Therefore, future studies will need to incorporate the site of progressive disease and the relative electric field strength at that location.

## Conflict of Interest

E. T. Wong received an unrestricted grant from Novocure for sponsored laboratory research. E. Lok and V. Hua report no disclosure.
